# [μ-1,3-Dioxo-1,3-bis(pyridin-2-yl)propane-2,2-diido-κ^2^ *N*,*C* ^2^:κ^2^ *C* ^2^,*N*′]bis[(1,3-diphenylpropane-1,3-dionato-κ^2^ *O*,*O*′)palladium(II)](*Pd*—*Pd*)

**DOI:** 10.1107/S1600536811017144

**Published:** 2011-05-14

**Authors:** Simona Maggini, Peter S. White

**Affiliations:** a Department of Chemistry, University of North Carolina, Chapel Hill, NC 27599-3290, USA.

## Abstract

The title compound, [Pd_2_(C_13_H_8_N_2_O_2_)(C_15_H_11_O_2_)_2_], crystallized from a mixture of ethanol and *n*-hexa­nes. The structure is the first example of β-diketonate in a dianionic κ^2^ *C*-coordination complex containing a Pd^II^—Pd^II^ bond. Both Pd^II^ atoms adopt a pseudo square-planar coordination geometry. The mol­ecular packing involves π-inter­actions between the phenyl rings of the 1,3-diphenyl­propane-1,3-dionato ligands with centroid–centroid distances in the range 3.823 (2)–3.868 (2) Å.

## Related literature

For related structures with rhodium, see: Herrmann *et al.* (1981 ▶, 1984 ▶), with mercury, see: McCandlish & Macklin (1975 ▶); Bonhomme *et al.* (1994 ▶); Toledano *et al.* (1994 ▶) and with germanium, tin and gold, see: Ganis *et al.* (1988 ▶); Djordjevic *et al.* (2003 ▶).
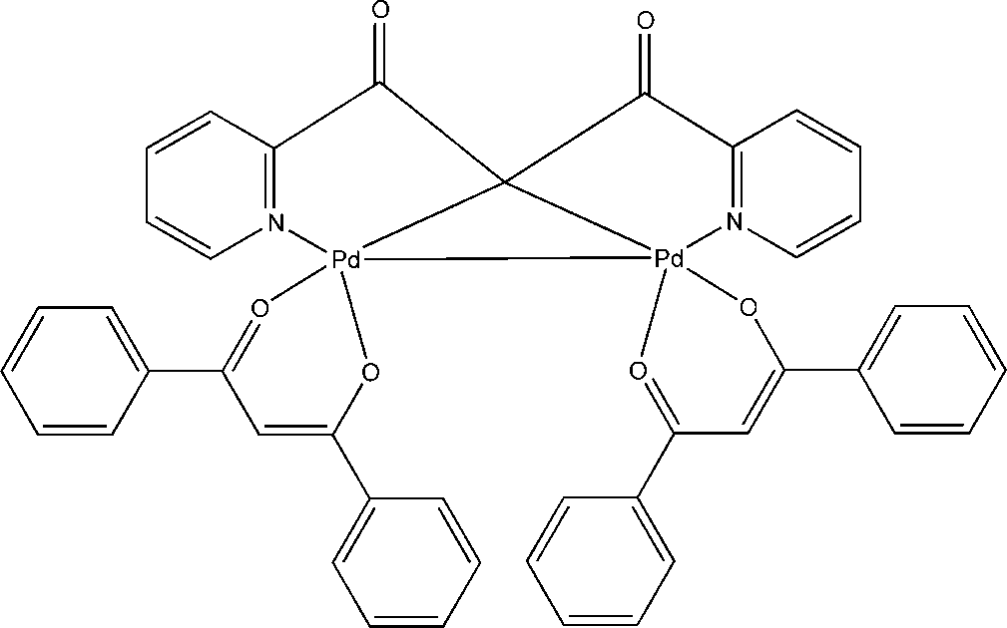


## Experimental

### 

#### Crystal data

[Pd_2_(C_13_H_8_N_2_O_2_)(C_15_H_11_O_2_)_2_]
*M* *_r_* = 883.49
Monoclinic, 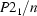

*a* = 15.2535 (5) Å
*b* = 10.6912 (4) Å
*c* = 20.9236 (7) Å
β = 96.498 (2)°
*V* = 3390.3 (2) Å^3^
*Z* = 4
Cu *K*α radiation
μ = 9.02 mm^−1^
*T* = 100 K
0.21 × 0.14 × 0.11 mm

#### Data collection

Bruker SMART APEXII CCD area-detector diffractometer
Absorption correction: numerical [*SADABS* (Bruker, 2004 ▶); *SORTAV* (Blessing, 1995 ▶)] *T*_min_ = 0.258, *T*_max_ = 0.451
21234 measured reflections
6139 independent reflections
5506 reflections with *I* > 2σ(*I*)
*R*_int_ = 0.033

#### Refinement

*R*[*F* ^2^ > 2σ(*F* ^2^)] = 0.030
*wR*(*F* ^2^) = 0.075
*S* = 1.07
6139 reflections
478 parameters
H-atom parameters constrained
Δρ_max_ = 0.81 e Å^−3^
Δρ_min_ = −0.51 e Å^−3^

### 

Data collection: *APEX2* (Bruker, 2004 ▶); cell refinement: *SAINT* (Bruker, 2004 ▶); data reduction: *SAINT*; program(s) used to solve structure: *SHELXS97* (Sheldrick, 2008 ▶); program(s) used to refine structure: *SHELXL97* (Sheldrick, 2008 ▶); molecular graphics: *ORTEP-3 for Windows* (Farrugia, 1997 ▶); software used to prepare material for publication: *WinGX* publication routines (Farrugia, 1999 ▶).

## Supplementary Material

Crystal structure: contains datablocks I, global. DOI: 10.1107/S1600536811017144/ff2010sup1.cif

Structure factors: contains datablocks I. DOI: 10.1107/S1600536811017144/ff2010Isup2.hkl

Additional supplementary materials:  crystallographic information; 3D view; checkCIF report

**Fig. 1.**
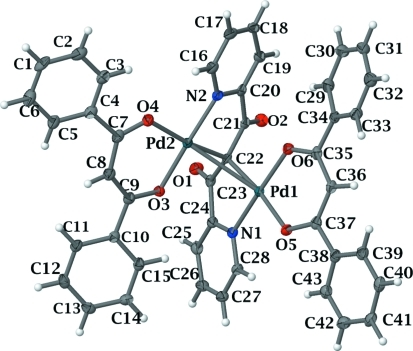
The molecular structure of [Pd2(C15H11O2)2(C13H8N2O2)]. Displacement ellipsoids are drawn at the 50% probability level.

Enhanced figure: interactive version of Fig. 1

## Figures and Tables

**Table 1:** Selected bond lengths (Å)

Pd1—N1	2.017 (3)
Pd1—O6	2.019 (2)
Pd1—C22	2.045 (3)
Pd1—O5	2.076 (2)
Pd1—Pd2	3.1056 (3)
Pd2—O3	2.013 (2)
Pd2—N2	2.016 (3)
Pd2—C22	2.051 (3)
Pd2—O4	2.063 (2)

## supplementary crystallographic information

### Comment

β-Diketonates are well known organic ligands, they play an important role in many research fields and applications. They are characterized by their ability to stabilize metallic fragments, form complexes with transition and main group elements in their neutral and anionic form, and assume different types of coordination modes (Herrmann *et al.*,1981; Herrmann *et al.*, 1984; McCandlish *et al.*, 1975; Bonhomme *et al.*, 1994; Toledano *et al.*, 1994; Ganis, *et al.*, 1988; Djordjevic *et al.*, 2003). There are several examples of β-diketonate complexes containing the ligand in its neutral or monoanionic form, while complexes containing β-diketonates in their dianionic form are rarer and only few of them have been structurally characterized. Here we report the first crystallographic characterization of a κ^2^*C*-bonded dianionic β-diketonate complex containing a Pd(II)—Pd(II) close interaction. Likely the rigidity of the bridging 1,3-di(pyridin-2-yl)propane-1,3-dionato ligand and the presence of its two N-donors contribute to stabilize the particular structure favoring a Pd(II)—Pd(II) proximity.

In the structure, the two palladium atoms adopt a pseudo square-planar coordination geometry (O—Pd—O angles of 91.65 (9)° and 91.42 (8)°; C—Pd—N of 82.90 (11)° and 82.49 (11)°. All the Pd—N, Pd—C and Pd—O bond lengths are in accord with their usual range values. The two palladiums present a similar environment, differentiated mostly by the Pd—O distances *trans* to carbon, which vary from 2.076 (2)Å to 2.063 (2) Å. Each 1,3-diphenylpropane-1,3-dionato ligand chelates one palladium, adopting its enolate form. Contrarily, the 1,3-di(pyridin-2-yl)propane-1,3-dionato possess well defined C=O double and C—C single bonds. The Pd(II)—Pd(II) distance is 3.1056 (3) Å. The Pd—C22—Pd and C22—Pd—Pd angles are respectively: 98.62 (13)° and 40.62 (8)°, 40.77 (8)°. The molecular packing involves π-interactions (centroid-centroid distances 3.823 (2) – 3.868 (2) Å) of the phenyl rings of the 1,3-diphenylpropane-1,3-dionato favored by an alternated up and down molecular disposition.

### Experimental

Bu_4_NOH 1*M* in MeOH (0.2 ml, 0.2 mmol) and [Zn(dppd)_2_(H_2_O)_2_] (dpd = 1,3-diphenylpropane-1,3-dione) (90 mg, 0.16 mmol) were added to a solution of Pd(CH_3_CN)_2_Cl_2_ (0.16 mmol) and 1,3-di(pyridin-2-yl)propane-1,3-dionato (0.16 mmol) in CH_2_Cl_2_ (12 ml). The resulting mixture was stirred at room temperature for 1 h. The solvent was removed and the red solid recovered was dissolved in ethanol. Crystals of [Pd(C_15_H_11_O_2_)_2_(C_13_H_8_N_2_O_2_)] were obtained by slow diffusion of n-hexanes into the previously prepared ethanol solution. The clear pink block-like crystals formed over a period of two weeks.

### Refinement

All non-hydrogen atoms were refined anisotropically. The hydrogen atoms were placed at their geometrically idealised positions and refined as riding with *U*_iso_(H) = 1.2*U*_eq_(C).

### Crystal data

**Table:**

[Pd_2_(C_13_H_8_N_2_O_2_)(C_15_H_11_O_2_)_2_]	*F*(000) = 1768
*M**_r_* = 883.49	*D*_x_ = 1.731 Mg m^−^^3^
Monoclinic, *P*2_1_/*n*	Cu *K*α radiation, λ = 1.54178 Å
Hall symbol: -P 2yn	Cell parameters from 9963 reflections
*a* = 15.2535 (5) Å	θ = 4.3–68.2°
*b* = 10.6912 (4) Å	µ = 9.02 mm^−^^1^
*c* = 20.9236 (7) Å	*T* = 100 K
β = 96.498 (2)°	Block-like, clear pink
*V* = 3390.3 (2) Å^3^	0.21 × 0.14 × 0.11 mm
*Z* = 4	

### Data collection

**Table:**

Bruker SMART APEXII CCD area-detector diffractometer	6139 independent reflections
Radiation source: Enhance (Cu) X-ray Source	5506 reflections with *I* > 2σ(*I*)
graphite	*R*_int_ = 0.033
φ and ω scans	θ_max_ = 69.2°, θ_min_ = 3.4°
Absorption correction: numerical [*SADABS* (Bruker, 2004); *SORTAV* (Blessing, 1995)]	*h* = −18→18
*T*_min_ = 0.258, *T*_max_ = 0.451	*k* = −12→12
21234 measured reflections	*l* = −25→24

### Refinement

**Table:**

Refinement on *F*^2^	Primary atom site location: structure-invariant direct methods
Least-squares matrix: full	Secondary atom site location: difference Fourier map
*R*[*F*^2^ > 2σ(*F*^2^)] = 0.030	Hydrogen site location: inferred from neighbouring sites
*wR*(*F*^2^) = 0.075	H-atom parameters constrained
*S* = 1.07	*w* = 1/[σ^2^(*F*_o_^2^) + (0.0409*P*)^2^ + 2.5899*P*] where *P* = (*F*_o_^2^ + 2*F*_c_^2^)/3
6139 reflections	(Δ/σ)_max_ = 0.002
478 parameters	Δρ_max_ = 0.81 e Å^−^^3^
0 restraints	Δρ_min_ = −0.51 e Å^−^^3^

### Special details

**Table:**

Geometry. All e.s.d.'s (except the e.s.d. in the dihedral angle between two l.s. planes) are estimated using the full covariance matrix. The cell e.s.d.'s are taken into account individually in the estimation of e.s.d.'s in distances, angles and torsion angles; correlations between e.s.d.'s in cell parameters are only used when they are defined by crystal symmetry. An approximate (isotropic) treatment of cell e.s.d.'s is used for estimating e.s.d.'s involving l.s. planes.
Refinement. Refinement of *F*^2^ against ALL reflections. The weighted *R*-factor *wR* and goodness of fit *S* are based on *F*^2^, conventional *R*-factors *R* are based on *F*, with *F* set to zero for negative *F*^2^. The threshold expression of *F*^2^ > σ(*F*^2^) is used only for calculating *R*-factors(gt) *etc*. and is not relevant to the choice of reflections for refinement. *R*-factors based on *F*^2^ are statistically about twice as large as those based on *F*, and *R*- factors based on ALL data will be even larger.

### Fractional atomic coordinates and isotropic or equivalent isotropic displacement parameters (Å^2^)

**Table:**

	*x*	*y*	*z*	*U*_iso_*/*U*_eq_	
Pd1	0.570612 (13)	0.61433 (2)	0.261676 (10)	0.01556 (7)	
Pd2	0.606750 (13)	0.68131 (2)	0.406689 (10)	0.01624 (7)	
O1	0.72762 (15)	0.3937 (2)	0.38650 (11)	0.0240 (5)	
O2	0.53896 (15)	0.3249 (2)	0.35850 (11)	0.0241 (5)	
O3	0.72555 (13)	0.7416 (2)	0.38639 (10)	0.0192 (5)	
O4	0.60128 (14)	0.8183 (2)	0.47560 (11)	0.0228 (5)	
O5	0.55041 (14)	0.7120 (2)	0.17549 (10)	0.0195 (5)	
O6	0.44018 (14)	0.6156 (2)	0.27046 (10)	0.0199 (5)	
N1	0.69998 (17)	0.5927 (2)	0.25269 (12)	0.0182 (5)	
N2	0.48873 (17)	0.6133 (3)	0.42465 (12)	0.0192 (6)	
C1	0.6179 (3)	1.1108 (3)	0.65876 (16)	0.0302 (8)	
H1	0.6072	1.1569	0.6959	0.036*	
C2	0.5509 (2)	1.0413 (3)	0.62577 (16)	0.0283 (8)	
H2	0.494	1.0406	0.6401	0.034*	
C3	0.5660 (2)	0.9730 (3)	0.57223 (15)	0.0239 (7)	
H3	0.5197	0.9243	0.5505	0.029*	
C4	0.6492 (2)	0.9746 (3)	0.54944 (15)	0.0208 (7)	
C5	0.7161 (2)	1.0456 (3)	0.58301 (15)	0.0247 (7)	
H5	0.7729	1.0479	0.5685	0.03*	
C6	0.7005 (3)	1.1131 (3)	0.63761 (17)	0.0310 (8)	
H6	0.7466	1.1606	0.6603	0.037*	
C7	0.6621 (2)	0.8976 (3)	0.49176 (15)	0.0206 (7)	
C8	0.7378 (2)	0.9144 (3)	0.45997 (15)	0.0228 (7)	
H8	0.774	0.9844	0.4727	0.027*	
C9	0.7652 (2)	0.8387 (3)	0.41170 (15)	0.0196 (7)	
C10	0.8509 (2)	0.8657 (3)	0.38645 (15)	0.0177 (6)	
C11	0.9201 (2)	0.9295 (3)	0.42223 (15)	0.0209 (7)	
H11	0.9117	0.9639	0.463	0.025*	
C12	1.0011 (2)	0.9427 (3)	0.39847 (16)	0.0235 (7)	
H12	1.048	0.9852	0.4233	0.028*	
C13	1.0137 (2)	0.8946 (3)	0.33912 (16)	0.0220 (7)	
H13	1.0694	0.9033	0.3233	0.026*	
C14	0.9450 (2)	0.8333 (3)	0.30213 (15)	0.0211 (7)	
H14	0.9533	0.8017	0.2608	0.025*	
C15	0.8647 (2)	0.8188 (3)	0.32613 (15)	0.0199 (7)	
H15	0.8181	0.7762	0.3011	0.024*	
C16	0.4325 (2)	0.6731 (3)	0.45877 (15)	0.0235 (7)	
H16	0.4439	0.7577	0.4709	0.028*	
C17	0.3579 (2)	0.6143 (4)	0.47686 (16)	0.0264 (8)	
H17	0.3177	0.659	0.4998	0.032*	
C18	0.3431 (2)	0.4908 (4)	0.46119 (15)	0.0267 (8)	
H18	0.2938	0.4482	0.4749	0.032*	
C19	0.4006 (2)	0.4283 (3)	0.42515 (15)	0.0225 (7)	
H19	0.3911	0.3431	0.4134	0.027*	
C20	0.4722 (2)	0.4936 (3)	0.40686 (14)	0.0191 (6)	
C21	0.5399 (2)	0.4372 (3)	0.36911 (14)	0.0181 (6)	
C22	0.60569 (19)	0.5277 (3)	0.34791 (13)	0.0163 (6)	
C23	0.69515 (19)	0.4714 (3)	0.34852 (14)	0.0171 (6)	
C24	0.7462 (2)	0.5184 (3)	0.29614 (14)	0.0188 (6)	
C25	0.8322 (2)	0.4823 (3)	0.29069 (16)	0.0240 (7)	
H25	0.8632	0.4294	0.322	0.029*	
C26	0.8717 (2)	0.5249 (3)	0.23869 (16)	0.0250 (7)	
H26	0.9303	0.5004	0.2334	0.03*	
C27	0.8257 (2)	0.6037 (3)	0.19421 (16)	0.0247 (7)	
H27	0.8528	0.6357	0.1589	0.03*	
C28	0.7392 (2)	0.6351 (3)	0.20222 (15)	0.0215 (7)	
H28	0.7069	0.6877	0.1714	0.026*	
C29	0.2731 (2)	0.5563 (3)	0.29292 (16)	0.0245 (7)	
H29	0.3203	0.5081	0.3137	0.029*	
C30	0.1895 (2)	0.5464 (3)	0.31316 (16)	0.0259 (7)	
H30	0.1798	0.4916	0.3474	0.031*	
C31	0.1207 (2)	0.6166 (3)	0.28318 (17)	0.0259 (7)	
H31	0.0638	0.6109	0.2972	0.031*	
C32	0.1347 (2)	0.6949 (3)	0.23298 (17)	0.0259 (7)	
H32	0.0871	0.7423	0.2121	0.031*	
C33	0.2177 (2)	0.7047 (3)	0.21288 (16)	0.0226 (7)	
H33	0.2267	0.759	0.1783	0.027*	
C34	0.2884 (2)	0.6356 (3)	0.24288 (15)	0.0188 (6)	
C35	0.3805 (2)	0.6491 (3)	0.22559 (15)	0.0184 (6)	
C36	0.3939 (2)	0.6962 (3)	0.16506 (15)	0.0194 (6)	
H36	0.3426	0.7093	0.1356	0.023*	
C37	0.4753 (2)	0.7263 (3)	0.14297 (15)	0.0191 (6)	
C38	0.4770 (2)	0.7789 (3)	0.07671 (15)	0.0204 (7)	
C39	0.4025 (2)	0.8285 (3)	0.04050 (16)	0.0241 (7)	
H39	0.3479	0.8302	0.0583	0.029*	
C40	0.4067 (2)	0.8753 (3)	−0.02085 (16)	0.0269 (8)	
H40	0.3557	0.9104	−0.0444	0.032*	
C41	0.4861 (2)	0.8705 (3)	−0.04777 (16)	0.0272 (8)	
H41	0.4892	0.9005	−0.0902	0.033*	
C42	0.5604 (2)	0.8218 (3)	−0.01227 (17)	0.0276 (8)	
H42	0.6148	0.8196	−0.0304	0.033*	
C43	0.5565 (2)	0.7762 (3)	0.04929 (16)	0.0241 (7)	
H43	0.608	0.743	0.073	0.029*	

### Atomic displacement parameters (Å^2^)

**Table:**

	*U*^11^	*U*^22^	*U*^33^	*U*^12^	*U*^13^	*U*^23^
Pd1	0.01383 (12)	0.02008 (13)	0.01257 (12)	−0.00005 (8)	0.00055 (8)	−0.00060 (8)
Pd2	0.01407 (12)	0.02168 (13)	0.01308 (12)	0.00017 (8)	0.00203 (8)	−0.00249 (9)
O1	0.0213 (11)	0.0311 (13)	0.0199 (12)	0.0073 (10)	0.0032 (9)	0.0035 (10)
O2	0.0229 (11)	0.0264 (13)	0.0232 (12)	−0.0018 (10)	0.0029 (9)	−0.0004 (10)
O3	0.0168 (10)	0.0227 (12)	0.0187 (11)	−0.0024 (9)	0.0050 (8)	−0.0040 (9)
O4	0.0198 (11)	0.0286 (13)	0.0203 (11)	−0.0013 (9)	0.0040 (9)	−0.0092 (10)
O5	0.0202 (11)	0.0232 (11)	0.0150 (11)	−0.0014 (9)	0.0011 (8)	0.0029 (9)
O6	0.0155 (10)	0.0269 (12)	0.0170 (11)	0.0025 (9)	0.0009 (8)	−0.0008 (9)
N1	0.0175 (13)	0.0222 (14)	0.0149 (13)	−0.0015 (11)	0.0015 (10)	−0.0029 (11)
N2	0.0158 (13)	0.0289 (15)	0.0129 (13)	0.0014 (11)	0.0015 (10)	0.0014 (11)
C1	0.047 (2)	0.0281 (19)	0.0159 (16)	0.0071 (16)	0.0066 (15)	−0.0057 (14)
C2	0.0357 (19)	0.0303 (19)	0.0203 (17)	0.0065 (16)	0.0087 (14)	0.0013 (14)
C3	0.0300 (17)	0.0233 (18)	0.0185 (16)	0.0021 (14)	0.0020 (13)	0.0007 (13)
C4	0.0253 (16)	0.0195 (16)	0.0176 (15)	0.0045 (13)	0.0025 (12)	0.0002 (13)
C5	0.0258 (17)	0.0284 (18)	0.0193 (16)	0.0024 (14)	0.0002 (13)	−0.0034 (14)
C6	0.038 (2)	0.030 (2)	0.0240 (18)	0.0035 (16)	−0.0026 (15)	−0.0070 (15)
C7	0.0226 (16)	0.0219 (16)	0.0168 (15)	0.0066 (13)	0.0000 (12)	0.0014 (13)
C8	0.0239 (16)	0.0250 (17)	0.0194 (16)	0.0000 (14)	0.0017 (13)	−0.0033 (14)
C9	0.0208 (15)	0.0211 (16)	0.0165 (15)	0.0016 (13)	−0.0001 (12)	0.0039 (13)
C10	0.0189 (15)	0.0179 (16)	0.0162 (15)	0.0009 (12)	0.0012 (12)	0.0006 (12)
C11	0.0277 (17)	0.0185 (16)	0.0164 (15)	−0.0016 (13)	0.0017 (13)	−0.0012 (13)
C12	0.0234 (16)	0.0188 (16)	0.0272 (17)	−0.0046 (13)	−0.0021 (13)	0.0010 (14)
C13	0.0195 (16)	0.0208 (17)	0.0261 (17)	0.0002 (13)	0.0051 (13)	0.0054 (13)
C14	0.0235 (16)	0.0228 (17)	0.0181 (16)	0.0003 (13)	0.0065 (13)	0.0017 (13)
C15	0.0220 (16)	0.0188 (16)	0.0180 (16)	0.0000 (13)	−0.0018 (12)	0.0012 (12)
C16	0.0219 (16)	0.0314 (19)	0.0170 (16)	0.0048 (14)	0.0020 (13)	−0.0012 (14)
C17	0.0188 (16)	0.044 (2)	0.0166 (16)	0.0075 (15)	0.0031 (13)	0.0035 (15)
C18	0.0157 (15)	0.048 (2)	0.0165 (16)	−0.0025 (15)	0.0012 (12)	0.0085 (15)
C19	0.0194 (15)	0.0304 (18)	0.0172 (15)	−0.0025 (14)	−0.0007 (12)	0.0074 (14)
C20	0.0176 (14)	0.0273 (18)	0.0113 (14)	0.0000 (13)	−0.0029 (11)	0.0024 (13)
C21	0.0185 (14)	0.0243 (18)	0.0107 (14)	0.0001 (13)	−0.0023 (11)	0.0022 (12)
C22	0.0172 (14)	0.0214 (16)	0.0100 (13)	0.0015 (12)	0.0008 (11)	−0.0022 (12)
C23	0.0176 (14)	0.0175 (15)	0.0157 (15)	0.0023 (12)	0.0001 (12)	−0.0027 (13)
C24	0.0176 (15)	0.0225 (17)	0.0165 (15)	−0.0019 (13)	0.0023 (12)	−0.0038 (13)
C25	0.0211 (16)	0.0264 (18)	0.0241 (17)	−0.0005 (14)	0.0000 (13)	−0.0033 (14)
C26	0.0177 (15)	0.0325 (19)	0.0259 (17)	−0.0007 (14)	0.0078 (13)	−0.0083 (15)
C27	0.0230 (17)	0.0317 (19)	0.0208 (17)	−0.0059 (14)	0.0084 (13)	−0.0067 (14)
C28	0.0222 (16)	0.0266 (18)	0.0154 (15)	−0.0046 (13)	0.0014 (12)	0.0008 (13)
C29	0.0219 (16)	0.0284 (18)	0.0221 (17)	0.0016 (14)	−0.0025 (13)	0.0041 (14)
C30	0.0213 (16)	0.0319 (19)	0.0245 (17)	−0.0017 (14)	0.0030 (13)	0.0059 (15)
C31	0.0181 (16)	0.035 (2)	0.0253 (18)	−0.0021 (14)	0.0044 (13)	−0.0020 (15)
C32	0.0197 (16)	0.0307 (19)	0.0266 (18)	0.0063 (14)	−0.0005 (13)	0.0015 (15)
C33	0.0233 (16)	0.0261 (18)	0.0182 (16)	0.0020 (14)	0.0013 (13)	0.0016 (13)
C34	0.0182 (15)	0.0215 (16)	0.0165 (15)	−0.0002 (13)	0.0011 (12)	−0.0049 (13)
C35	0.0182 (15)	0.0158 (15)	0.0206 (16)	0.0018 (12)	−0.0006 (12)	−0.0043 (12)
C36	0.0197 (15)	0.0205 (16)	0.0171 (15)	0.0011 (13)	−0.0016 (12)	−0.0007 (13)
C37	0.0232 (16)	0.0152 (15)	0.0184 (15)	0.0006 (12)	0.0000 (12)	−0.0023 (12)
C38	0.0259 (16)	0.0157 (15)	0.0189 (15)	−0.0030 (13)	−0.0002 (12)	−0.0024 (13)
C39	0.0275 (17)	0.0224 (17)	0.0221 (17)	−0.0021 (14)	0.0013 (13)	−0.0041 (14)
C40	0.0344 (19)	0.0234 (18)	0.0209 (17)	−0.0038 (15)	−0.0056 (14)	−0.0004 (14)
C41	0.043 (2)	0.0223 (18)	0.0153 (16)	−0.0079 (15)	0.0007 (15)	−0.0002 (13)
C42	0.0348 (19)	0.0265 (18)	0.0226 (17)	−0.0059 (15)	0.0083 (14)	−0.0014 (14)
C43	0.0271 (17)	0.0235 (17)	0.0208 (16)	−0.0020 (14)	−0.0014 (13)	−0.0005 (14)

### Geometric parameters (Å, °)

**Table:**

Pd1—N1	2.017 (3)	C16—H16	0.95
Pd1—O6	2.019 (2)	C17—C18	1.374 (5)
Pd1—C22	2.045 (3)	C17—H17	0.95
Pd1—O5	2.076 (2)	C18—C19	1.391 (5)
Pd1—Pd2	3.1056 (3)	C18—H18	0.95
Pd2—O3	2.013 (2)	C19—C20	1.386 (4)
Pd2—N2	2.016 (3)	C19—H19	0.95
Pd2—C22	2.051 (3)	C20—C21	1.496 (4)
Pd2—O4	2.063 (2)	C21—C22	1.496 (4)
O1—C23	1.216 (4)	C22—C23	1.490 (4)
O2—C21	1.221 (4)	C23—C24	1.501 (4)
O3—C9	1.285 (4)	C24—C25	1.384 (4)
O4—C7	1.274 (4)	C25—C26	1.380 (5)
O5—C37	1.273 (4)	C25—H25	0.95
O6—C35	1.283 (4)	C26—C27	1.386 (5)
N1—C24	1.345 (4)	C26—H26	0.95
N1—C28	1.350 (4)	C27—C28	1.390 (5)
N2—C16	1.338 (4)	C27—H27	0.95
N2—C20	1.348 (4)	C28—H28	0.95
C1—C6	1.382 (5)	C29—C34	1.387 (5)
C1—C2	1.384 (5)	C29—C30	1.392 (5)
C1—H1	0.95	C29—H29	0.95
C2—C3	1.378 (5)	C30—C31	1.382 (5)
C2—H2	0.95	C30—H30	0.95
C3—C4	1.406 (5)	C31—C32	1.378 (5)
C3—H3	0.95	C31—H31	0.95
C4—C5	1.395 (5)	C32—C33	1.383 (5)
C4—C7	1.492 (4)	C32—H32	0.95
C5—C6	1.394 (5)	C33—C34	1.397 (5)
C5—H5	0.95	C33—H33	0.95
C6—H6	0.95	C34—C35	1.496 (4)
C7—C8	1.408 (5)	C35—C36	1.399 (4)
C8—C9	1.394 (5)	C36—C37	1.410 (4)
C8—H8	0.95	C36—H36	0.95
C9—C10	1.493 (4)	C37—C38	1.499 (4)
C10—C15	1.396 (4)	C38—C39	1.397 (5)
C10—C11	1.400 (4)	C38—C43	1.399 (5)
C11—C12	1.390 (5)	C39—C40	1.386 (5)
C11—H11	0.95	C39—H39	0.95
C12—C13	1.378 (5)	C40—C41	1.393 (5)
C12—H12	0.95	C40—H40	0.95
C13—C14	1.394 (5)	C41—C42	1.385 (5)
C13—H13	0.95	C41—H41	0.95
C14—C15	1.385 (5)	C42—C43	1.385 (5)
C14—H14	0.95	C42—H42	0.95
C15—H15	0.95	C43—H43	0.95
C16—C17	1.389 (5)		
			
N1—Pd1—O6	173.80 (10)	C17—C18—C19	119.6 (3)
N1—Pd1—C22	82.49 (11)	C17—C18—H18	120.2
O6—Pd1—C22	94.87 (10)	C19—C18—H18	120.2
N1—Pd1—O5	91.56 (9)	C20—C19—C18	118.2 (3)
O6—Pd1—O5	91.42 (8)	C20—C19—H19	120.9
C22—Pd1—O5	172.94 (10)	C18—C19—H19	120.9
N1—Pd1—Pd2	92.87 (7)	N2—C20—C19	122.0 (3)
O6—Pd1—Pd2	88.70 (6)	N2—C20—C21	114.2 (3)
C22—Pd1—Pd2	40.77 (8)	C19—C20—C21	123.7 (3)
O5—Pd1—Pd2	136.47 (6)	O2—C21—C22	125.2 (3)
O3—Pd2—N2	177.23 (10)	O2—C21—C20	119.8 (3)
O3—Pd2—C22	94.36 (10)	C22—C21—C20	115.0 (3)
N2—Pd2—C22	82.90 (11)	C23—C22—C21	112.4 (3)
O3—Pd2—O4	91.65 (9)	C23—C22—Pd1	109.8 (2)
N2—Pd2—O4	91.04 (10)	C21—C22—Pd1	115.92 (19)
C22—Pd2—O4	171.58 (11)	C23—C22—Pd2	111.9 (2)
O3—Pd2—Pd1	86.11 (6)	C21—C22—Pd2	107.45 (19)
N2—Pd2—Pd1	92.03 (7)	Pd1—C22—Pd2	98.62 (13)
C22—Pd2—Pd1	40.62 (8)	O1—C23—C22	125.9 (3)
O4—Pd2—Pd1	146.06 (7)	O1—C23—C24	120.0 (3)
C9—O3—Pd2	124.4 (2)	C22—C23—C24	114.1 (3)
C7—O4—Pd2	124.9 (2)	N1—C24—C25	122.6 (3)
C37—O5—Pd1	124.1 (2)	N1—C24—C23	114.5 (3)
C35—O6—Pd1	124.1 (2)	C25—C24—C23	122.8 (3)
C24—N1—C28	118.8 (3)	C26—C25—C24	118.4 (3)
C24—N1—Pd1	116.6 (2)	C26—C25—H25	120.8
C28—N1—Pd1	124.0 (2)	C24—C25—H25	120.8
C16—N2—C20	119.3 (3)	C25—C26—C27	119.8 (3)
C16—N2—Pd2	124.5 (2)	C25—C26—H26	120.1
C20—N2—Pd2	115.7 (2)	C27—C26—H26	120.1
C6—C1—C2	119.9 (3)	C26—C27—C28	118.8 (3)
C6—C1—H1	120.1	C26—C27—H27	120.6
C2—C1—H1	120.1	C28—C27—H27	120.6
C3—C2—C1	120.6 (3)	N1—C28—C27	121.6 (3)
C3—C2—H2	119.7	N1—C28—H28	119.2
C1—C2—H2	119.7	C27—C28—H28	119.2
C2—C3—C4	120.6 (3)	C34—C29—C30	121.0 (3)
C2—C3—H3	119.7	C34—C29—H29	119.5
C4—C3—H3	119.7	C30—C29—H29	119.5
C5—C4—C3	118.2 (3)	C31—C30—C29	119.7 (3)
C5—C4—C7	123.5 (3)	C31—C30—H30	120.2
C3—C4—C7	118.3 (3)	C29—C30—H30	120.2
C6—C5—C4	120.7 (3)	C32—C31—C30	120.1 (3)
C6—C5—H5	119.6	C32—C31—H31	120
C4—C5—H5	119.6	C30—C31—H31	120
C1—C6—C5	120.0 (3)	C31—C32—C33	120.2 (3)
C1—C6—H6	120	C31—C32—H32	119.9
C5—C6—H6	120	C33—C32—H32	119.9
O4—C7—C8	124.8 (3)	C32—C33—C34	120.7 (3)
O4—C7—C4	115.0 (3)	C32—C33—H33	119.6
C8—C7—C4	120.1 (3)	C34—C33—H33	119.6
C9—C8—C7	126.7 (3)	C29—C34—C33	118.3 (3)
C9—C8—H8	116.7	C29—C34—C35	119.0 (3)
C7—C8—H8	116.7	C33—C34—C35	122.6 (3)
O3—C9—C8	127.0 (3)	O6—C35—C36	126.8 (3)
O3—C9—C10	113.6 (3)	O6—C35—C34	113.8 (3)
C8—C9—C10	119.4 (3)	C36—C35—C34	119.4 (3)
C15—C10—C11	118.5 (3)	C35—C36—C37	127.1 (3)
C15—C10—C9	118.5 (3)	C35—C36—H36	116.4
C11—C10—C9	122.8 (3)	C37—C36—H36	116.4
C12—C11—C10	120.3 (3)	O5—C37—C36	124.7 (3)
C12—C11—H11	119.8	O5—C37—C38	115.5 (3)
C10—C11—H11	119.8	C36—C37—C38	119.8 (3)
C13—C12—C11	120.3 (3)	C39—C38—C43	118.4 (3)
C13—C12—H12	119.8	C39—C38—C37	123.1 (3)
C11—C12—H12	119.8	C43—C38—C37	118.5 (3)
C12—C13—C14	120.2 (3)	C40—C39—C38	121.2 (3)
C12—C13—H13	119.9	C40—C39—H39	119.4
C14—C13—H13	119.9	C38—C39—H39	119.4
C15—C14—C13	119.5 (3)	C39—C40—C41	119.7 (3)
C15—C14—H14	120.3	C39—C40—H40	120.2
C13—C14—H14	120.3	C41—C40—H40	120.2
C14—C15—C10	121.1 (3)	C42—C41—C40	119.6 (3)
C14—C15—H15	119.4	C42—C41—H41	120.2
C10—C15—H15	119.4	C40—C41—H41	120.2
N2—C16—C17	121.6 (3)	C43—C42—C41	120.8 (3)
N2—C16—H16	119.2	C43—C42—H42	119.6
C17—C16—H16	119.2	C41—C42—H42	119.6
C18—C17—C16	119.2 (3)	C42—C43—C38	120.3 (3)
C18—C17—H17	120.4	C42—C43—H43	119.8
C16—C17—H17	120.4	C38—C43—H43	119.8
			
N1—Pd1—Pd2—O3	26.32 (10)	C18—C19—C20—C21	178.9 (3)
O6—Pd1—Pd2—O3	−159.68 (9)	N2—C20—C21—O2	169.4 (3)
C22—Pd1—Pd2—O3	101.33 (14)	C19—C20—C21—O2	−7.6 (4)
O5—Pd1—Pd2—O3	−68.98 (11)	N2—C20—C21—C22	−9.8 (4)
N1—Pd1—Pd2—N2	−151.63 (11)	C19—C20—C21—C22	173.2 (3)
O6—Pd1—Pd2—N2	22.37 (10)	O2—C21—C22—C23	−34.5 (4)
C22—Pd1—Pd2—N2	−76.62 (15)	C20—C21—C22—C23	144.7 (3)
O5—Pd1—Pd2—N2	113.07 (11)	O2—C21—C22—Pd1	92.8 (3)
N1—Pd1—Pd2—C22	−75.01 (15)	C20—C21—C22—Pd1	−88.0 (3)
O6—Pd1—Pd2—C22	98.99 (14)	O2—C21—C22—Pd2	−158.0 (2)
O5—Pd1—Pd2—C22	−170.31 (15)	C20—C21—C22—Pd2	21.1 (3)
N1—Pd1—Pd2—O4	113.48 (13)	N1—Pd1—C22—C23	−13.8 (2)
O6—Pd1—Pd2—O4	−72.51 (13)	O6—Pd1—C22—C23	160.6 (2)
C22—Pd1—Pd2—O4	−171.50 (16)	Pd2—Pd1—C22—C23	−117.1 (3)
O5—Pd1—Pd2—O4	18.18 (14)	N1—Pd1—C22—C21	−142.4 (2)
C22—Pd2—O3—C9	−178.0 (2)	O6—Pd1—C22—C21	31.9 (2)
O4—Pd2—O3—C9	−3.9 (2)	Pd2—Pd1—C22—C21	114.3 (3)
Pd1—Pd2—O3—C9	142.2 (2)	N1—Pd1—C22—Pd2	103.31 (12)
O3—Pd2—O4—C7	−2.2 (3)	O6—Pd1—C22—Pd2	−82.32 (11)
N2—Pd2—O4—C7	177.1 (3)	O3—Pd2—C22—C23	36.6 (2)
Pd1—Pd2—O4—C7	−87.7 (3)	N2—Pd2—C22—C23	−143.0 (2)
N1—Pd1—O5—C37	160.3 (2)	Pd1—Pd2—C22—C23	115.5 (3)
O6—Pd1—O5—C37	−14.3 (2)	O3—Pd2—C22—C21	160.42 (19)
Pd2—Pd1—O5—C37	−103.9 (2)	N2—Pd2—C22—C21	−19.19 (19)
C22—Pd1—O6—C35	−172.5 (2)	Pd1—Pd2—C22—C21	−120.7 (2)
O5—Pd1—O6—C35	10.7 (2)	O3—Pd2—C22—Pd1	−78.84 (11)
Pd2—Pd1—O6—C35	147.2 (2)	N2—Pd2—C22—Pd1	101.55 (12)
C22—Pd1—N1—C24	11.0 (2)	C21—C22—C23—O1	−34.0 (4)
O5—Pd1—N1—C24	−172.8 (2)	Pd1—C22—C23—O1	−164.5 (3)
Pd2—Pd1—N1—C24	50.5 (2)	Pd2—C22—C23—O1	87.0 (3)
C22—Pd1—N1—C28	−177.4 (3)	C21—C22—C23—C24	145.8 (3)
O5—Pd1—N1—C28	−1.2 (3)	Pd1—C22—C23—C24	15.2 (3)
Pd2—Pd1—N1—C28	−137.9 (2)	Pd2—C22—C23—C24	−93.2 (3)
C22—Pd2—N2—C16	−172.3 (3)	C28—N1—C24—C25	−0.8 (5)
O4—Pd2—N2—C16	13.6 (3)	Pd1—N1—C24—C25	171.3 (2)
Pd1—Pd2—N2—C16	−132.6 (2)	C28—N1—C24—C23	−177.1 (3)
C22—Pd2—N2—C20	15.8 (2)	Pd1—N1—C24—C23	−5.0 (3)
O4—Pd2—N2—C20	−158.4 (2)	O1—C23—C24—N1	172.6 (3)
Pd1—Pd2—N2—C20	55.4 (2)	C22—C23—C24—N1	−7.2 (4)
C6—C1—C2—C3	0.7 (5)	O1—C23—C24—C25	−3.7 (5)
C1—C2—C3—C4	−1.2 (5)	C22—C23—C24—C25	176.5 (3)
C2—C3—C4—C5	1.0 (5)	N1—C24—C25—C26	0.3 (5)
C2—C3—C4—C7	179.2 (3)	C23—C24—C25—C26	176.2 (3)
C3—C4—C5—C6	−0.1 (5)	C24—C25—C26—C27	1.1 (5)
C7—C4—C5—C6	−178.3 (3)	C25—C26—C27—C28	−1.8 (5)
C2—C1—C6—C5	0.2 (5)	C24—N1—C28—C27	0.1 (5)
C4—C5—C6—C1	−0.4 (5)	Pd1—N1—C28—C27	−171.4 (2)
Pd2—O4—C7—C8	8.1 (4)	C26—C27—C28—N1	1.3 (5)
Pd2—O4—C7—C4	−171.0 (2)	C34—C29—C30—C31	−0.2 (5)
C5—C4—C7—O4	165.2 (3)	C29—C30—C31—C32	0.8 (5)
C3—C4—C7—O4	−12.9 (4)	C30—C31—C32—C33	−0.8 (5)
C5—C4—C7—C8	−14.0 (5)	C31—C32—C33—C34	0.1 (5)
C3—C4—C7—C8	167.8 (3)	C30—C29—C34—C33	−0.4 (5)
O4—C7—C8—C9	−8.8 (5)	C30—C29—C34—C35	176.2 (3)
C4—C7—C8—C9	170.3 (3)	C32—C33—C34—C29	0.5 (5)
Pd2—O3—C9—C8	4.7 (5)	C32—C33—C34—C35	−176.0 (3)
Pd2—O3—C9—C10	−177.73 (19)	Pd1—O6—C35—C36	−3.6 (4)
C7—C8—C9—O3	1.6 (6)	Pd1—O6—C35—C34	177.06 (19)
C7—C8—C9—C10	−175.8 (3)	C29—C34—C35—O6	−19.8 (4)
O3—C9—C10—C15	22.7 (4)	C33—C34—C35—O6	156.6 (3)
C8—C9—C10—C15	−159.5 (3)	C29—C34—C35—C36	160.7 (3)
O3—C9—C10—C11	−153.3 (3)	C33—C34—C35—C36	−22.8 (5)
C8—C9—C10—C11	24.5 (5)	O6—C35—C36—C37	−5.9 (5)
C15—C10—C11—C12	−1.5 (5)	C34—C35—C36—C37	173.5 (3)
C9—C10—C11—C12	174.5 (3)	Pd1—O5—C37—C36	10.6 (4)
C10—C11—C12—C13	0.8 (5)	Pd1—O5—C37—C38	−169.15 (19)
C11—C12—C13—C14	0.6 (5)	C35—C36—C37—O5	1.7 (5)
C12—C13—C14—C15	−1.4 (5)	C35—C36—C37—C38	−178.6 (3)
C13—C14—C15—C10	0.7 (5)	O5—C37—C38—C39	−164.2 (3)
C11—C10—C15—C14	0.7 (5)	C36—C37—C38—C39	16.0 (5)
C9—C10—C15—C14	−175.4 (3)	O5—C37—C38—C43	17.0 (4)
C20—N2—C16—C17	0.6 (5)	C36—C37—C38—C43	−162.8 (3)
Pd2—N2—C16—C17	−171.0 (2)	C43—C38—C39—C40	−0.6 (5)
N2—C16—C17—C18	2.2 (5)	C37—C38—C39—C40	−179.5 (3)
C16—C17—C18—C19	−2.8 (5)	C38—C39—C40—C41	1.4 (5)
C17—C18—C19—C20	0.7 (5)	C39—C40—C41—C42	−1.5 (5)
C16—N2—C20—C19	−2.8 (4)	C40—C41—C42—C43	0.9 (5)
Pd2—N2—C20—C19	169.6 (2)	C41—C42—C43—C38	−0.1 (5)
C16—N2—C20—C21	−179.9 (3)	C39—C38—C43—C42	0.0 (5)
Pd2—N2—C20—C21	−7.5 (3)	C37—C38—C43—C42	178.8 (3)
C18—C19—C20—N2	2.1 (4)		
